# Intestinal Helminth Infections in Ghanaian Children from the Ashanti Region between 2007 and 2008—A Retrospective Cross-Sectional Real-Time PCR-Based Assessment

**DOI:** 10.3390/tropicalmed7110374

**Published:** 2022-11-14

**Authors:** Charity Wiafe Akenten, Felix Weinreich, Ellis Kobina Paintsil, John Amuasi, Dennis Fosu, Ulrike Loderstädt, Jürgen May, Hagen Frickmann, Denise Dekker

**Affiliations:** 1 Kumasi Centre for Collaborative Research in Tropical Medicine (KCCR), South-End, Asuogya Road, Kumasi 039-5028, Ghana; 2 Department of Microbiology and Hospital Hygiene, Bundeswehr Hospital Hamburg, 20359 Hamburg, Germany; 3 Department of Hospital Hygiene & Infectious Diseases, University Medicine Göttingen, 37075 Göttingen, Germany; 4 Department Infectious Disease Epidemiology, Bernhard Nocht Institute for Tropical Medicine Hamburg, 20359 Hamburg, Germany; 5 German Center for Infection Research (DZIF), Partner Site Hamburg-Lübeck-Borstel-Riems, 20359 Hamburg, Germany; 6 Tropical Medicine II, University Medical Center Hamburg-Eppendorf (UKE), 20251 Hamburg, Germany; 7 Department of Microbiology, Virology and Hygiene, University Medicine Rostock, 18057 Rostock, Germany; 8 Research Group One Health Bacteriology, Bernhard Nocht Institute for Tropical Medicine Hamburg, 20359 Hamburg, Germany

**Keywords:** helminth, epidemiology, diarrhea, *Ascaris*, hookworm, *Strongyloides*, *Trichuris*, *Taenia*, *Schistosoma*, *Hymenolepis*, *Enterobius*, Ghana

## Abstract

In spite of ongoing eradication programs, helminth infections are still a medical issue in Ghana. For follow-up assessments on the decline of regional helminth infections, historic baseline prevalence values obtained with standardized diagnostic procedures can be helpful. In this retrospective cross-sectional study, real-time PCR targeting the nematodes *Ancylostoma* spp. (ITS2), *Ascaris lumbricoides* (ITS1), *Enterobius vermicularis* (ITS1), *Necator americanus* (ITS2), *Strongyloides stercoralis* (18S rRNA) and *Trichuris trichiura* (18S rRNA), the trematodes *Schistosoma* spp. (ITS2) as well as the cestodes *Hymenolepis nana* (ITS1), *Taenia saginata* (ITS1) and *Taenia solium* (ITS1) was applied with 2046 DNA eluates from stool samples of Ghanaian children from the Ashanti region collected between 2007 and 2008 in order to retrospectively define prevalence values. The overall prevalence was low with 3.8% (*n* = 77) and only 0.1% (*n* = 2) double infections with helminths were recorded. The three most frequently detected enteric helminth species comprised 2% *S. stercoralis* (*n* = 41), 0.8% *H. nana* (*n* = 16), and 0.7% *N. americanus* (*n* = 14), while only sporadic infection events were recorded for other helminth species comprising 0.1% *E. vermicularis* (*n* = 2), 0.1% *Schistosoma* spp. (*n* = 2), 0.1% *T. saginata* (*n* = 1) and 0.1% *T. trichiura* (*n* = 1). *A. lumbricoides*, *Ancylostoma* spp. and *T. solium* were not detected at all. In conclusion, the retrospective assessment suggests a low prevalence of enteric helminth infections in Ghanaian children from the Ashanti Region within the assessment period between 2007 and 2008.

## 1. Introduction

Intestinal helminth infections are common, particularly in resource-limited tropical settings [1,2] where access even to baseline hygiene precautions such as hand washing with soap is sometimes scarcely available [3]. In addition, meta-analyses have suggested the association of specific helminth infections with age, sex, co-infections, previous treatment, and lifestyle [4,5]. In the tropics, co-infections with different helminths as well as co-infections of helminths and other severe infections are quite frequently observed, making mutual supportive interactions likely [6,7,8,9,10]. In contrast, the worm burden declines in settings showing socio-economic development where systematic deworming programs are implemented [11].

From the public health perspective, deworming programs are useful because intestinal helminth infections have been reported to be associated with stunted growth, cognitive impairment [12,13], likely effects even on adult productivity [14] as well as with pregnancy and birth complications [15,16]. As a complication of hookworm infections in Ghana, resistance determinants against benzimidazoles are common [17], which might partially explain the varying effectiveness of benzimidazole-based treatment as observed in Ghanaian patients [18,19].

For West African Ghana, the prevalence of multiple helminth infections has been reported. In Ghanaian individuals, infection rates with intestinal helminths have been shown to be in the range of 2–22% [20,21,22,23] with declining prevalence over recent decades [24], urogenital schistosomiasis in the range of 2.5–12% [20,24], while intestinal schistosomiasis was regionally reported for more than 90% of assessed Ghanaian children [25] but in less than 2% for other Ghanaian patients [22]. Swimming in surface water is an independent risk factor for schistosomiasis in Ghana [26,27], and reinfection rates are high with up to 40% within six months in some areas [28]. In historic assessments covering the previous decades, very high infection rates >50% were quite common for schistosomiasis in Ghana [29,30,31,32,33]. In exposed individuals such as waste handlers, prevalence rates for soil-transmissible helminths of 5% have been reported [34], and prevalence rates >20% were observed in farmers [35]. In the case of Ghanaian farmers, wastewater irrigation increases the risk of intestinal helminth infections by factor 3 [36]. Cases of taeniasis have been detected in Ghana including cerebral affections [21,37] same as trematode infections with *Fasciola gigantica* [38] and *Dicrocoelium dendriticum* [39]. Even solitary egg findings suggesting infections with small liver flukes like *Clonorchis* spp. or *Opisthorchis* spp. have been reported from Ghana [40]. Frequently detected intestinal helminths in Ghana comprise *Ascaris lumbricoides*, hookworms with higher infection rates for *Necator americanus* than for *Ancylostoma* spp., *Hymenolepis* spp., *Taenia* spp., *Strongyloides stercoralis*, *Schistosoma* spp. and *Trichuris trichiura* [21,22,23,41,42,43]. In spite of declining overall helminth infection rates, intestinal helminths are yet among the top five outpatient morbidities in Ghana [44], making Ghana a suitable site to study inference between helminth infections and other diseases like, e.g., allergic diatheses [45]. Consequently, modeling suggested that Ghana is among the countries where the interruption of transmission of soil-transmitted helminths may become challenging [46]. Animal reservoirs are elements of the transmission cycles as well [47].

To follow up with the decline of helminth infections in Ghana [44] with the aim of final eradication, information on historic prevalence values determined with up-to-date real-time PCR approaches, which were not available yet when the samples were collected, can be helpful to define baseline prevalence values. Accordingly, enteric helminth-specific real-time PCR from frozen residual DNA eluate samples derived from stool specimens of children with and without diarrhea collected in the Ghanaian Ashanti region between 2007 and 2008 [48,49,50,51,52,53] was performed in order to contribute to the available epidemiologic knowledge on historic regional intestinal helminth prevalence.

## 2. Materials and Methods

### 2.1. Study Type and Sample Collection

For the retrospective cross-sectional assessment, residual nucleic acid extractions from 2046 stool samples which were collected in the course of a study from 2007 till 2008 from Ghanaian children from the Ashanti Region with and without clinical diarrhea (defined by ≥3 unformed stools per day) were included in the assessment. As detailed elsewhere, multiple screenings for viral, bacterial, and protozoan enteropathogens were performed with those samples [48,49,50,51,52,53], while molecular helminth assessment had not yet been conducted so far. Nucleic acid extraction was performed with the QiaAMP DNA Stool Mini Kit (Qiagen, Hilden, Germany) as described by the manufacturer. Subsequently, the samples were stored frozen at −80 °C. As reported previously [48,49,50,51,52,53], children were ≤13 years of age with a median of less than 3 years in a left-shifted distribution, while the proportions of boys and girls as well as the proportions of individuals with and without diarrhea were nearly evenly distributed. Further, about one out of five children were diagnosed with malaria at the time of the assessment.

### 2.2. Applied Real-Time PCRs for the Detection of Helminth DNA in Stool Samples, Inclusion and Exclusion Criteria and Statistical Assessment

All nucleic acid extractions from human stool were subjected to in-house multiplex real-time PCR targeting *Ascaris lumbricoides* (ITS1, minimum detectable genomic equivalent: 1.3 × 10^2^), *Ancylostoma* spp. (ITS2, minimum detectable genomic equivalent: 1.3 × 10^2^), *Enterobius vermicularis* (ITS1, minimum detectable genomic equivalent: 1.6 × 10^1^), *Hymenolepis nana* (ITS1, minimum detectable genomic equivalent: 1.4 × 10^1^), *Necator americanus* (ITS2, minimum detectable genomic equivalent: 1.3 × 10^2^), *Schistosoma* spp. (detecting *S. haematobium*, *S. mansoni* and *S. intercalatum* without discrimination on the species level, ITS2, minimum detectable genomic equivalent: 3.0 × 10^0^), *Strongyloides stercoralis* (18S rRNA, minimum detectable genomic equivalent: 1.3 × 10^2^), *Taenia saginata* (ITS1, minimum detectable genomic equivalent: 9.0 × 10^0^), *Taenia solium* (ITS1, minimum detectable genomic equivalent: 1.3 × 10^1^), and *Trichuris trichiura* (18S rRNA, minimum detectable genomic equivalent: 1.1 × 10^1^), respectively. Plasmid-based positive controls and PCR-grade water-based negative controls were included in each real-time PCR run. The sequences of the primer and probe oligonucleotides as well as of the positive control plasmid inserts as published elsewhere [54] are shown in Appendix A Table A1. The real-time PCRs were performed on RotorGene Q thermocyclers exactly as described elsewhere; performance characteristics of the assays have been provided there as well [54]. Based on the experience of a previous multicentric evaluation study [54] and participation in the international external laboratory assessment scheme for helminth PCR [55], late real-time PCR signals with cycle threshold (Ct) values higher than 40 with typical sigmoid-shaped amplification curves still indicate specific amplification. All samples, for which sufficient residual nucleic acid material was available, were included in the assessment. The study samples were treated in the same way as patient samples in diagnostic routine use of the applied helminth PCR assays without technical replicates. There were no exclusion criteria. The results were descriptively demonstrated without further statistical analyses.

### 2.3. Ethics

Ethical clearance for the sample collection and the informed consent procedure was obtained from the Committee on Human Research, Publications and Ethics, School of Medical Science, Kwame Nkrumah University of Science and Technology, Kumasi, Ghana (reference CHRPE/KNUST/KATH/01_10_08). To be included in the study, written informed consent was obtained from the parents or the legal guardian prior to the enrolment. In case of non-participation, medical treatment was nevertheless provided. Further, anonymous characterization of residual samples was granted by the medical association of Hamburg, Germany, (reference number: WF-011/19, obtained on 11 March 2019). The assessments were performed in line with the Declaration of Helsinki and its amendments.

## 3. Results

**Figure 1:**
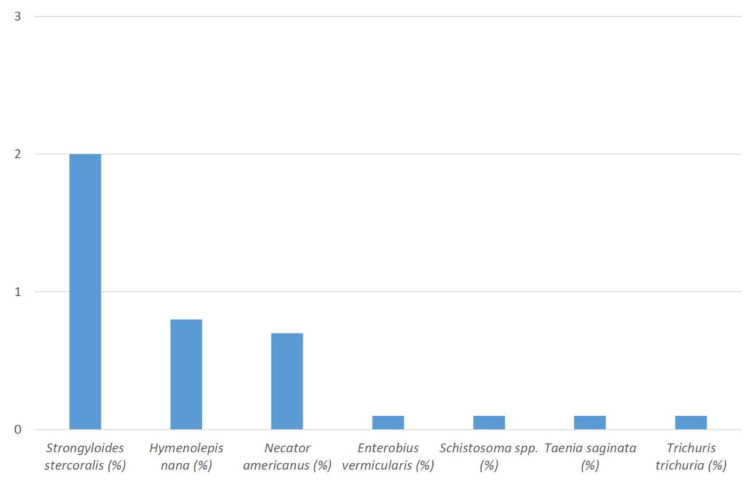
Percentages (%) of recorded helminth DNA in the stool samples of the study participants.

From a total of 2046 included residual sample materials, positive real-time PCR results were obtained from 3.8% (*n* = 77) samples. Prevalence values for detected target DNA of the different assessed helminths ranged from 0.0% (*n* = 0) to 2.0% (*n* = 41). Prevalence values > 0.5% were recorded for only three species with 2.0% *Strongyloides stercoralis* (*n* = 41), 0.8% *Hymenolepis nana* (*n* = 16), and 0.7% *Necator americanus* (*n* = 14). Solely individual cases were observed for other helminths with 0.1% *Enterobius vermicularis* (*n* = 2), 0.1% *Schistosoma* spp. (*n* = 2), 0.1% *Taenia saginata* (*n* = 1), and 0.1% *Trichuris trichiura* (*n* = 1). No cases at all were seen for *Ascaris lumbricoides*, *Ancylostoma* spp. and *Taenia solium* (Figure 1). Details including the recorded cycle threshold (Ct value) ranges are provided in Table 1. In 2.6% (2/77) of the positive samples and thus in 0.1% (2/2046) of the totally assessed samples, co-infections with different helminths were recorded. The co-infections comprised two different target helminths each, i.e., *T. trichiura* and *T. saginata* in one case as well as *N. americanus* and *S. stercoralis* in the other case.

Focusing on associations of helminth infections and malaria, the co-incidence of helminth infections and malaria was 22.7% (14/77). Helminth infections co-occurring with malaria comprised *S. stercoralis* (*n* = 6), *H. nana* (*n* = 5), *E. vermicularis* (*n* = 1), *N. americanus* (*n* = 1), and a co-infection with *S. stercoralis* and *N. americanus* (*n* = 1). A minority of 24.7% (19/77) of the recorded helminth infections was associated with reported diarrhea, comprising *S. stercoralis* (*n* = 12), *H. nana* (*n* = 3), *N. americanus* (*n* = 3), and *Schistosoma* spp. (*n* = 1). No significant differences were observed between cycle threshold (Ct) values of helminth infections in patients with and without diarrhea (Table 2).

## 4. Discussion

A PCR-based assessment of helminth prevalences was performed with stool samples of Ghanaian children from the Ashanti Region. Residual sample material was used that was collected in the years 2007 and 2008 [48,49,50,51,52,53]. Therefore, baseline prevalence values for follow-up assessments were established. In line with ongoing intervention programs in Ghana [56], the overall prevalence of recorded helminth infections was low. In detail, the epidemiological coverage of anti-helminthic mass drug administration for Ghanaian pre-school children was estimated to be 98.37% in the study year 2008 [57], likely explaining the very low detection rates in the stool samples. Although socioeconomic and behavioral aspects specifically related to helminth infections had not been systematically recorded for the study population, malnourishment of no more than 10% and vaccination rates ranging between 80% and more than 90% as reported elsewhere [48] suggest little hints for neglect and a good general access of the assessed children to the country’s public health infrastructure.

In contrast to the low prevalence values as observed in the study here, estimates of the helminth prevalence on a Pan-African level in the decade of the study period were much higher. In a review from 2009, Sub-Saharan African prevalence estimates for enteric infections with hookworms, *A. lumbricoides* and *T. trichiura* but also for infections with *Schistosoma* spp. were higher than 20% each [58]. Another research group [6] argued that those estimates might have been too high, suggesting lower prevalence estimates of 16.5% for hookworms, 6.6% for *A. lumbricoides,* and 4.4% for *T. trichiura* instead. Still, those estimates were much higher than the proportions of infections observed in the assessed Ghanaian children.

In line with previous assessments in Ghana [20,21,22,23,42,43], nematodes such as *S. stercoralis* and hookworms quantitatively dominated. In comparison, the very low rate of *Schistosoma* spp. was less expected [25,59] but reflects the scattered distribution of *S. mansoni* as reported for Ghana [22,60,61]. Interestingly, the cestode *H. nana* was the second most frequent helminth within the assessed Ghanaian stool samples, although its prevalence was still low and well in line with previous scarcely available Ghanaian studies including this parameter [21,62]. Real-time PCR-based screening for *H. nana* is yet rarely applied in epidemiological studies compared to more frequently used assays targeting nematodes [54]. The protocol from this study was first introduced after evaluation in 2020 [54]. All other helminths included in the screening were only rarely identified or absent. Helminth co-infections, i.e., infections with more than one helminth species, were observed in two instances (0.1%) only.

Due to the very low overall detection rates, assessment of associations with demographic features or clinical features was not possible. It should be noted that such associations are difficult to interpret because of the high rates of co-infections with facultative enteropathogenic bacteria and protozoa [48,49]. While only a minority of 19 helminth infections with a distribution resembling the overall distribution of positive helminth real-time PCR results in this study was associated with reported diarrhea, a total of 27 co-infections with the bacterial and protozoan pathogens *Campylobacter jejuni* (*n* = 10), *Giardia duodenalis* (*n* = 7), *Shigella* spp./enteroinvasive *Escherichia coli* (not further discriminated, *n* = 6), *Cryptosporidium parvum* (*n* = 3) and *Salmonella enterica* (*n* = 1) had been previously detected in the same 19 samples [48]. Accordingly, any etiological relevance of the helminth detections with a focus on diarrhea is highly questionable for the donors of the respective stool samples, which is also in line with the seemingly paradox, non-significant finding of higher mean Ct values for *H. nana* and *N. americanus* in samples of patients with diarrhea compared to patients without diarrhea. In a similar way, the proportion of helminth detections in stool samples of patients with malaria just matched the overall proportion of malaria cases within the assessed population, not allowing for any further conclusions.

Interestingly, the abundance of the helminth species *S. stercoralis* and *N. americanus* in the assessed samples outnumbered orally transmitted helminths. The specific reasons are unknown because previous assessments of enteric pathogens other than helminths in the study population suggested frequent transmission events via the oral route [48,49]. So, it is likely that the finding more reflects a generally higher regional abundance of these species rather than a lower relevance of the oral transmission route.

The observed low abundance of *Enterobius vermicularis* is surprising in a cohort consisting of children. It remains unclear whether this finding was just a consequence of the Ghanaian anti-helminthic mass drug administration program [57]. Alternatively, sensitivity issues of the real-time PCR-based testing approach might have also played a role here, because scotch tape preparations were not performed but target DNA was just amplified from stool DNA extractions.

The study has a number of limitations. First, storage of the nucleic acid eluates for about 13 years since the time of sample collection may have resulted in minor nucleic acid degradation, potentially resulting in decreased sensitivity with regard to samples with a priori low target DNA concentrations close to the technical detection limits of the real-time PCRs. To keep the probability of this type of bias low, the nucleic acids within the eluates had been optimally preserved by storing the samples deep frozen at −80 °C. Moreover, the recorded cycle threshold values were in the typical range as observed for infected individuals and sufficiently far away from the detection threshold, suggesting that the DNA was still widely intact. In addition, DNA preservation had been exemplarily controlled in the course of a recent test comparison assessment [63]. For the respective study [63], selected residual samples had been re-assessed with the same real-time PCR assays for DNA of enteric protozoa and entero-invasive bacteria which had also been applied with the same stool samples shortly after acquisition in Ghana. Obtained cycle threshold values had been in a comparable range, thus suggesting that deep freezing-based DNA preservation had been successful. Second, no microscopic results were available for correlation and confirmation of the PCR results. While real-time PCR from stool samples is a priori more sensitive than microscopy for protozoan pathogens, this is considerably less unambiguously true in the case of helminths [64], from which nucleic acids are more difficult to extract from eggs and cuticula cells [65]. So, microscopic results would have provided true additional value but the retrospective design of the study made this option unfeasible. Fourth, the study did not provide a comprehensive assessment of all helminth infections potentially occurring in Ghanaian individuals. For example, no serological screening for toxocariasis was conducted, although high seroprevalence rates have previously been reported from Ghana [66]. Fifth, the conducted stool assessment for *Schistosoma* spp. DNA did not exclude the shedding of *Schistosoma haematobium* eggs via the patients’ urine and so, it did not provide a comprehensive overview of schistosomiasis in the assessed Ghanaian population. Sixth, lacking systematic assessment of demographic, socioeconomic, and behavioral data related to helminth infections limits the interpretability of the study results.

## 5. Conclusions

In spite of the abovementioned limitations, the results of the study suggested a low overall infection rate of the assessed Ghanaian children from the Ashanti Region in 2007 and 2008 with enteric helminths. Next to the expected dominance of the nematodes *S. stercoralis* and *N. americanus*, the cestode *H. nana* was among the most frequently identified helminths. The assessment provides a small piece to the epidemiological puzzle and baseline values for future follow-up assessments in this geographic region.

## Figures and Tables

**Table 1:** Positive PCR results and recorded cycle threshold (Ct) value ranges. A total of 2046 samples were assessed.

Target Pathogen	Number of Positives (*n*)	Proportion of Positives (%)	Minimum Recorded Ct Value	Maximum Recorded Ct Value	Mean Ct Value	Standard Deviation (SD)	Median Ct Value
*Ascaris lumbricoides*	0	0	n.a.	n.a.	n.a.	n.a.	n.a.
*Ancylostoma* spp.	0	0	n.a.	n.a.	n.a.	n.a.	n.a.
*Enterobius vermicularis*	2	0.1	27.0	33.0	30.0	3.0	30.0
*Hymenolepis nana*	16	0.8	19.0	35.0	28.7	4.4	31.0
*Necator americanus*	14	0.7	27.0	41.2	34.0	3.4	34.0
*Schistosoma* spp.	2	0.1	18.0	24.0	21.0	3.0	21.0
*Strongyloides stercoralis*	41	2.0	23.0	44.0	30.3	5.0	30.3
*Taenia saginata*	1	0.1	28.0	28.0	28.0	n.a.	28.0
*Taenia solium*	0	0	n.a.	n.a.	n.a.	n.a.	n.a.
*Trichuris trichiura*	1	0.1	28.0	28.0	28.0	n.a.	28.0

n.a. = not applicable.

**Table 2:** Ct values in case of helminth infections in patients with and without reported diarrhea.

Helminth	Mean Ct Value from Samples of Patients with Diarrhea (± Standard Deviation SD)	Mean Ct Value from Samples of Patients without Diarrhea (± Standard Deviation SD)	Significance *p* *
*Strongyloides stercoralis*	30.0 (6.5)	30.2 (4.5)	*p* = 0.73 (n.s.)
*Hymenolepis nana*	30.7 (3.2)	28.2 (4.8)	*p* = 0.28 (n.s.)
*Necator americanus*	37.0 (4.6)	33.3 (3.1)	*p* = 0.21 (n.s.)
*Schistosoma* spp.	18 (-)	24 (-)	n.e.

* Calculated with Mann–Whitney U-testing applying the software GraphPad Instat version 3.06 (GraphPad Software Inc., La Jolla, CA, USA). spp. = species (plural). n.e. = not estimable. n.s. = not significant.

## Data Availability

All relevant data are provided within the manuscript. Raw data can be made available on reasonable request.

## Appendix A

**Table A1:** Sequences of the primer and probe oligonucleotides (hybridization probes including the used reporter and quencher molecules) as well as of the positive control plasmid inserts of the applied helminth-specific real-time PCRs. The positive control plasmid inserts were included in pEX-A128 vector backbones (Eurofins Scientific SE, Luxembourg).

*Ascaris lumbricoides*-specific real-time PCR oligonucleotides	
forward primer	5′-GTAATAGCAGTCGGCGGTTTCTT-3′
reverse primer	5′-GCCCAACATGCCACCTATTC-3′
probe	5′-ROX-TTGGCGGACAATTGCATGCGAT-BHQ2-3′
positive control insert	5′-GGTGATGTAATAGCAGTCGGCGGTTTCTTTTTTTTTGGCGGACAATTGCATGCGATTTGCTATGTGTTGAGGGAGAATAGGTGGCATGTTGGGCTTGTTA-3′
*Strongyloides stercoralis*-specific real-time PCR oligonucleotides	
forward primer	5′-GAATTCCAAGTAAACGTAAGTCATTAGC-3′
reverse primer	5′-TGCCTCTGGATATTGCTCAGTTC-3′
probe	5′-CY5-ACACACCGGCCGTCGCTGC-BHQ2-3′
positive control insert	5′-AACGAGGAATTCCAAGTAAACGTAAGTCATTAGCTTACATTGATTACGTCCCTGCCCTTTGTACACACCGGCCGTCGCTGCCCGGAACTGAGCAATATCCAGAGGCAGGAAGA-3′
*Ancyclostoma* spp.-specific real-time PCR oligonucleotides	
forward primer	5′-GAATGACAGCAAACTCGTTGTTG-3′
reverse primer	5′-ATACTAGCCACTGCCGAAACGT-3′
probe	5′-YAKYE-ATCGTTTACCGACTTTAG-MGBEQ-3′
positive control insert	5′-TGCGCTGAATGACAGCAAACTCGTTGTTGCTGCTGAATCGTTTACCGACTTTAGAACGTTTCGGCAGTGGCTAGTATAACAAC-3′
*Necator americanus*-specific real-time PCR oligonucleotides	
forward primer	5′-CTGTTTGTCGAACGGTACTTGC-3′
reverse primer	5′-ATAACAGCGTGCACATGTTGC-3′
probe	5′-FAM-CTGTACTACGCATTGTATAC-MGBEQ-3′
positive control insert	5′-GAACACTGTTTGTCGAACGGTACTTGCTCTGTACTACGCATTGTATACGTGTTCAGCAATTCCCGTTTAAGTGAAGAACACACGTGCAACATGTGCACGCTGTTATTCACTACG-3′
*Trichuris trichiura*-specific real-time PCR oligonucleotides	
forward primer	5′-TTGAAACGACTTGCTCATCAACTT-3′
reverse primer	5′-CTGATTCTCCGTTAACCGTTGTC-3′
probe	5′-YAKYE-CGATGGTACGCTACGTGCTTACCATGG-MGBEQ-3′
positive control insert	5′-CGACGATGCTTTGAAACGACTTGCTCATCAACTTTCGATGGTACGCTACGTGCTTACCATGGTGACAACGGTTAACGGAGAATCAGGGTTCGGCTC-3′
*Schistosoma* spp.-specific real-time PCR oligonucleotides	
forward primer	5′-GGTCTAGATGACTTGATYGAGATGCT-3′
reverse primer	5′-TCCCGAGCGYGTATAATGTCATTA-3′
probe	5′-FAM-TGGGTTGTGCTCGAGTCGTGGC-BHQ1-3′
positive control insert	5′-TAGTCTGGTCTAGATGACTTGATTGAGATGCTGCGGTGGGTTGTGCTCGAGTCGTGGCTTAATGACATTATACACGCTCGGGATAATTC-3′
*Taenia solium*-specific real-time PCR oligonucleotides	
forward primer	5′-ATGGATCAATCTGGGTGGAGTT-3′
reverse primer	5′-ATCGCAGGGTAAGAAAAGAAGGT-3′
probe	5′-Cy5-TGGTACTGCTGTGGCGGCGG-BHQ2-3′
positive control insert	5′-TTGACTGATGATGGATCAATCTGGGTGGAGTTGGTGGTACTGCTGTGGCGGCGGTATTGTCAACTTCTTCTGTACCTTCTTTTCTTACCCTGCGATGGGGTGCCTA-3′
*Taenia saginata*-specific real-time PCR oligonucleotides	
forward primer	5′-GCGTCGTCTTTGCGTTACAC-3′
reverse primer	5′-TGACACAACCGCGCTCTG-3′
probe	5′-ROX-CCACAGCACCAGCGACAGCAGCAA-BHQ2-3′
positive control insert	5′-GCCCCATCATGCGTCGTCTTTGCGTTACACGTGGCGATGTTGCTGCTGTCGCTGGTGCTGTGGTGGCGGCGCAGAGCGCGGTTGTGTCACCGTTGGTGG-3′
*Enterobius vermicularis*-specific real-time PCR oligonucleotides	
forward primer	5′CGGTGTAATTTTGTTGGTGTCTATG-3′
reverse primer	5′-TGGCAGCATTGCAAACTAATG-3′
probe	5′-FAM-TGTGCCAGTCAACGCCTAAACCGT-C-BHQ1-3′
positive control insert	5′-TGTAATATAACGGTGTAATTTTGTTGGTGTCTATGCTTTGTGCCAGTCAACGCCTAAACCGTCGTTGATGTGTGTATAAGATGAAGCATAAAGCAAAAGGTTTGCTACTTGTAGCAGA-CTAGACTTAATAAGCATTAGTTTGCAATGCTGCCAACTATGATAA-3′
*Hymenolepis nana*-specific real-time PCR oligonucleotides	
forward primer	5′-CATTGTGTACCAAATTGATGATGAGTA-3′
reverse primer	5′-CAACTGACAGCATGTTTCGATATG-3′
probe	5′-JOE-CGTGTGCGCCTCTGGCTTACCG-BHQ1-3′
positive control insert	5′-ACACTTATTACATTGTGTACCAAATTGATGATGAGTAGACGTGTGCGCCTCTGGCTTACCGTTTACTGCCTCGTCATATCGAAACATGCTGTCAGTTGCTGCTGCTCA-3′

## References

[1] Ananthakrishnan S., Nalini P., Pani S.P. (1997). Intestinal geohelminthiasis in the developing world. Natl. Med. J. India.

[2] Markell E.K. (1985). Intestinal nematode infections. Pediatr. Clin. N. Am..

[3] Shrestha A., Schindler C., Odermatt P., Gerold J., Erismann S., Sharma S., Koju R., Utzinger J., Cissé G. (2018). Intestinal parasite infections and associated risk factors among schoolchildren in Dolakha and Ramechhap districts, Nepal: A cross-sectional study. Parasit. Vectors.

[4] Wright J.E., Werkman M., Dunn J.C., Anderson R.M. (2018). Current epidemiological evidence for predisposition to high or low intensity human helminth infection: A systematic review. Parasit. Vectors.

[5] Anderson R.M. (1986). The population dynamics and epidemiology of intestinal nematode infections. Trans. R. Soc. Trop. Med. Hyg..

[6] Karagiannis-Voules D.A., Biedermann P., Ekpo U.F., Garba A., Langer E., Mathieu E., Midzi N., Mwinzi P., Polderman A.M., Raso G. (2015). Spatial and temporal distribution of soil-transmitted helminth infection in sub-Saharan Africa: A systematic review and geostatistical meta-analysis. Lancet Infect. Dis..

[7] Basavaraju S.V., Schantz P. (2006). Soil-transmitted helminths and Plasmodium falciparum malaria: Epidemiology, clinical manifestations, and the role of nitric oxide in malaria and geohelminth co-infection. Do worms have a protective role in *P. falciparum* infection?. Mt. Sinai. J. Med..

[8] Eziefula A.C., Brown M. (2008). Intestinal nematodes: Disease burden, deworming and the potential importance of co-infection. Curr. Opin. Infect. Dis..

[9] Yatich N.J., Yi J., Agbenyega T., Turpin A., Rayner J.C., Stiles J.K., Ellis W.O., Funkhouser E., Ehiri J.E., Williams J.H. (2009). Malaria and intestinal helminth co-infection among pregnant women in Ghana: Prevalence and risk factors. Am. J. Trop. Med. Hyg..

[10] Hartgers F.C., Obeng B.B., Boakye D., Yazdanbakhsh M. (2008). Immune responses during helminth-malaria co-infection: A pilot study in Ghanaian school children. Parasitology.

[11] Donohue R.E., Cross Z.K., Michael E. (2019). The extent, nature, and pathogenic consequences of helminth polyparasitism in humans: A meta-analysis. PLoS Negl. Trop. Dis..

[12] McCarty T.R., Turkeltaub J.A., Hotez P.J. (2014). Global progress towards eliminating gastrointestinal helminth infections. Curr. Opin. Gastroenterol..

[13] Watkins W.E., Pollitt E. (1997). “Stupidity or worms”: Do intestinal worms impair mental performance?. Psychol. Bull..

[14] Guyatt H. (2000). Do intestinal nematodes affect productivity in adulthood?. Parasitol. Today.

[15] Yatich N.J., Jolly P.E., Funkhouser E., Agbenyega T., Rayner J.C., Ehiri J.E., Turpin A., Stiles J.K., Ellis W.O., Jiang Y. (2010). The effect of malaria and intestinal helminth coinfection on birth outcomes in Kumasi, Ghana. Am. J. Trop. Med. Hyg..

[16] Roberts T., Gravett C.A., Velu P.P., Theodoratou E., Wagner T.A., Zhang J.S., Campbell H., Rubens C.E., Gravett M.G., Rudan I. (2011). Epidemiology and aetiology of maternal parasitic infections in low- and middle-income countries. J. Glob. Health.

[17] Orr A.R., Quagraine J.E., Suwondo P., George S., Harrison L.M., Dornas F.P., Evans B., Caccone A., Humphries D., Wilson M.D. (2019). Genetic Markers of Benzimidazole Resistance among Human Hookworms (*Necator americanus*) in Kintampo North Municipality, Ghana. Am. J. Trop. Med. Hyg..

[18] Humphries D., Nguyen S., Kumar S., Quagraine J.E., Otchere J., Harrison L.M., Wilson M., Cappello M. (2017). Effectiveness of Albendazole for Hookworm Varies Widely by Community and Correlates with Nutritional Factors: A Cross-Sectional Study of School-Age Children in Ghana. Am. J. Trop. Med. Hyg..

[19] Humphries D., Simms B.T., Davey D., Otchere J., Quagraine J., Terryah S., Newton S., Berg E., Harrison L.M., Boakye D. (2013). Hookworm infection among school age children in Kintampo north municipality, Ghana: Nutritional risk factors and response to albendazole treatment. Am. J. Trop. Med. Hyg..

[20] Orish V.N., Ofori-Amoah J., Amegan-Aho K.H., Osisiogu E.U., Osei-Yeboah J., Lokpo S.Y., Allotey E.A., Adu-Amankwaah J., Azuma D.E., Agordoh P.D. (2021). Eosinophilia in school-going children with Plasmodium falciparum and helminth infections in the Volta Region of Ghana. Pan. Afr. Med. J..

[21] Adu-Gyasi D., Asante K.P., Frempong M.T., Gyasi D.K., Iddrisu L.F., Ankrah L., Dosoo D., Adeniji E., Agyei O., Gyaase S. (2018). Epidemiology of soil transmitted Helminth infections in the middle-belt of Ghana, Africa. Parasite Epidemiol. Control.

[22] Cunningham L.J., Odoom J., Pratt D., Boatemaa L., Asante-Ntim N., Attiku K., Banahene B., Osei-Atweneboana M., Verweij J.J., Molyneux D. (2018). Expanding molecular diagnostics of helminthiasis: Piloting use of the GPLN platform for surveillance of soil transmitted helminthiasis and schistosomiasis in Ghana. PLoS Negl. Trop. Dis..

[23] Mirisho R., Neizer M.L., Sarfo B. (2017). Prevalence of Intestinal Helminths Infestation in Children Attending Princess Marie Louise Children’s Hospital in Accra, Ghana. J. Parasitol. Res..

[24] Ayeh-Kumi P.F., Addo-Osafo K., Attah S.K., Tetteh-Quarcoo P.B., Obeng-Nkrumah N., Awuah-Mensah G., Abbey H.N., Forson A., Cham M., Asare L. (2016). Malaria, helminths and malnutrition: A cross-sectional survey of school children in the South-Tongu district of Ghana. BMC Res. Notes.

[25] Armoo S., Cunningham L.J., Campbell S.J., Aboagye F.T., Boampong F.K., Hamidu B.A., Osei-Atweneboana M.Y., Stothard J.R., Adams E.R. (2020). Detecting Schistosoma mansoni infections among pre-school-aged children in southern Ghana: A diagnostic comparison of urine-CCA, real-time PCR and Kato-Katz assays. BMC Infect. Dis..

[26] Kulinkina A.V., Kosinski K.C., Adjei M.N., Osabutey D., Gyamfi B.O., Biritwum N.K., Bosompem K.M., Naumova E.N. (2019). Contextualizing Schistosoma haematobium transmission in Ghana: Assessment of diagnostic techniques and individual and community water-related risk factors. Acta Trop..

[27] Kosinski K.C., Kulinkina A.V., Tybor D., Osabutey D., Bosompem K.M., Naumova E.N. (2016). Agreement among Four Prevalence Metrics for Urogenital Schistosomiasis in the Eastern Region of Ghana. Biomed. Res. Int..

[28] Kulinkina A.V., Walz Y., Koch M., Biritwum N.K., Utzinger J., Naumova E.N. (2018). Improving spatial prediction of Schistosoma haematobium prevalence in southern Ghana through new remote sensors and local water access profiles. PLoS Negl. Trop. Dis..

[29] Tetteh I.K., Adjei R.O., Sasu S., Appiah-Kwakye L. (2004). Index of potential contamination: *Schistosoma haematobium* infections in school children in the Ashanti Region of Ghana. East Afr. Med. J..

[30] Klumpp R.K., Webbe G. (1987). Focal, seasonal and behavioural patterns of infection and transmission of *Schistosoma haematobium* in a farming village at the Volta Lake, Ghana. J. Trop. Med. Hyg..

[31] Scott D., Senker K., England E.C. (1982). Epidemiology of human *Schistosoma haematobium* infection around Volta Lake, Ghana, 1973-75. Bull. World Health Organ..

[32] Lyons G.R. (1974). Schistosomiasis in north-western Ghana. Bull. World Health Organ..

[33] Bozdĕch V. (1973). Das Vorkommen von Schistosoma haematobium (Bilharz) und *Schistosoma mansoni* (Sambon) in städtische Populationen von Accra-Ghana und Kaduna-Nigeria [The incidence of *Schistosoma haematobium* (Bilharz) and *Schistosoma mansoni* (Sambon) in urban populations of Accra-Ghana and of Kaduna-Nigeria (author’s transl)]. Zentralbl. Bakteriol. Orig. A.

[34] Kretchy J.P., Dzodzomenyo M., Ayi I., Dwomoh D., Agyabeng K., Konradsen F., Dalsgaard A. (2021). The Incidence, Intensity, and Risk Factors for Soil Transmissible Helminthes Infections among Waste Handlers in a Large Coastal Periurban Settlement in Southern Ghana. J. Environ. Public Health.

[35] Squire S.A., Yang R., Robertson I., Ayi I., Squire D.S., Ryan U. (2018). Gastrointestinal helminths in farmers and their ruminant livestock from the Coastal Savannah zone of Ghana. Parasitol, Res..

[36] Amoah I.D., Abubakari A., Stenström T.A., Abaidoo R.C., Seidu R. (2016). Contribution of Wastewater Irrigation to Soil Transmitted Helminths Infection among Vegetable Farmers in Kumasi, Ghana. PLoS Negl. Trop. Dis..

[37] Zoli A., Shey-Njila O., Assana E., Nguekam J.P., Dorny P., Brandt J., Geerts S. (2003). Regional status, epidemiology and impact of *Taenia solium* cysticercosis in Western and Central Africa. Acta Trop..

[38] Addy F., Romig T., Wassermann M. (2018). Genetic characterisation of *Fasciola gigantica* from Ghana. Vet. Parasitol. Reg. Stud. Rep..

[39] Ofori M., Bogoch I.I., Ephraim R.K. (2015). Prevalence of *Dicrocoelium dendriticum* ova in Ghanaian school children. J. Trop. Pediatr..

[40] Asare K.K., Boampong J.N., Ameyaw E.O., Thomford A.K., Afoakwah R., Kwakye-Nuako G., Thomford K.P., Quashie N.B. (2014). Microscopic identification of possible *Clonorchis*/*Opisthorchis* infection in two Ghanaian women with undiagnosed abdominal discomfort: Two case reports. J. Med. Case Rep..

[41] Boyko R.H., Harrison L.M., Humphries D., Galvani A.P., Townsend J.P., Otchere J., Wilson M.D., Cappello M. (2020). Dogs and pigs are transport hosts of *Necator americanus*: Molecular evidence for a zoonotic mechanism of human hookworm transmission in Ghana. Zoonoses Public Health.

[42] Egbi G., Steiner-Asiedu M., Kwesi F.S., Ayi I., Ofosu W., Setorglo J., Klobodu S.S., Armar-Klemesu M. (2014). Anaemia among school children older than five years in the Volta Region of Ghana. Pan Afr. Med. J..

[43] Bakker H. (1969). Ancylostomiasis in the Dormaa area, Ghana. Trop. Geogr. Med..

[44] Osei F.B., Stein A. (2017). Spatio-temporal analysis of small-area intestinal parasites infections in Ghana. Sci. Rep..

[45] Amoah A.S., Boakye D.A., Yazdanbakhsh M., van Ree R. (2017). Influence of Parasitic Worm Infections on Allergy Diagnosis in Sub-Saharan Africa. Curr. Allergy Asthma Rep..

[46] Brooker S.J., Nikolay B., Balabanova D., Pullan R.L. (2015). Global feasibility assessment of interrupting the transmission of soil-transmitted helminths: A statistical modelling study. Lancet Infect. Dis..

[47] Agyei A.D. (1991). Epidemiological observations on helminth infections of calves in southern Ghana. Trop. Anim. Health Prod..

[48] Krumkamp R., Sarpong N., Schwarz N.G., Adlkofer J., Loag W., Eibach D., Hagen R.M., Adu-Sarkodie Y., Tannich E., May J. (2015). Gastrointestinal infections and diarrheal disease in Ghanaian infants and children: An outpatient case-control study. PLoS Negl. Trop. Dis..

[49] Eibach D., Krumkamp R., Hahn A., Sarpong N., Adu-Sarkodie Y., Leva A., Käsmaier J., Panning M., May J., Tannich E. (2016). Application of a multiplex PCR assay for the detection of gastrointestinal pathogens in a rural African setting. BMC Infect. Dis..

[50] Eibach D., Krumkamp R., Al-Emran H.M., Sarpong N., Hagen R.M., Adu-Sarkodie Y., Tannich E., May J. (2015). Molecular characterization of *Cryptosporidium* spp. among children in rural Ghana. PLoS Negl. Trop. Dis..

[51] Leva A., Eibach D., Krumkamp R., Käsmaier J., Rubbenstroth D., Adu-Sarkodie Y., May J., Tannich E., Panning M. (2016). Diagnostic performance of the Luminex xTAG gastrointestinal pathogens panel to detect rotavirus in Ghanaian children with and without diarrhoea. Virol. J..

[52] Graul S., Böttcher S., Eibach D., Krumkamp R., Käsmaier J., Adu-Sarkodie Y., May J., Tannich E., Panning M. (2017). High diversity of human parechovirus including novel types in stool samples from Ghanaian children. J. Clin. Virol..

[53] Vinnemeier C.D., Klupp E.M., Krumkamp R., Rolling T., Fischer N., Owusu-Dabo E., Addo M.M., Adu-Sarkodie Y., Käsmaier J., Aepfelbacher M. (2016). *Tropheryma whipplei* in children with diarrhoea in rural Ghana. Clin. Microbiol. Infect..

[54] Köller T., Hahn A., Altangerel E., Verweij J.J., Landt O., Kann S., Dekker D., May J., Loderstädt U., Podbielski A. (2020). Comparison of commercial and in-house real-time PCR platforms for 15 parasites and microsporidia in human stool samples without a gold standard. Acta Trop..

[55] Cools P., van Lieshout L., Koelewijn R., Addiss D., Ajjampur S.S.R., Ayana M., Bradbury R.S., Cantera J.L., Dana D., Fischer K. (2020). First international external quality assessment scheme of nucleic acid amplification tests for the detection of Schistosoma and soil-transmitted helminths, including Strongyloides: A pilot study. PLoS Negl. Trop. Dis..

[56] Ahiadorme M., Morhe E. (2020). Soil transmitted helminth infections in Ghana: A ten year review. Pan. Afr. Med. J..

[57] Harhay M.O., Horton J., Olliaro P.L. (2010). Epidemiology and control of human gastrointestinal parasites in children. Expert Rev. Anti. Infect. Ther..

[58] Hotez P.J., Kamath A. (2009). Neglected tropical diseases in sub-saharan Africa: Review of their prevalence, distribution, and disease burden. PLoS Negl. Trop. Dis..

[59] Anyan W.K., Abonie S.D., Aboagye-Antwi F., Tettey M.D., Nartey L.K., Hanington P.C., Anang A.K., Muench S.B. (2019). Concurrent *Schistosoma mansoni* and *Schistosoma haematobium* infections in a peri-urban community along the Weija dam in Ghana: A wake up call for effective National Control Programme. Acta Trop..

[60] Wen S.T., Chu K.Y. (1984). Preliminary schistosomiasis survey in the lower Volta River below Akosombo Dam, Ghana. Ann. Trop. Med. Parasitol..

[61] Amankwa J.A., Bloch P., Meyer-Lassen J., Olsen A., Christensen N.O. (1994). Urinary and intestinal schistosomiasis in the Tono Irrigation Scheme, Kassena/Nankana District, upper east region, Ghana. Trop. Med. Parasitol..

[62] Abaka-Yawson A., Sosu S.Q., Kwadzokpui P.K., Afari S., Adusei S., Arko-Mensah J. (2020). Prevalence and Determinants of Intestinal Parasitic Infections among Pregnant Women Receiving Antenatal Care in Kasoa Polyclinic, Ghana. J. Environ. Public Health.

[63] Weinreich F., Hahn A., Eberhardt K.A., Kann S., Köller T., Warnke P., Dupke S., Dekker D., May J., Frickmann H. (2022). Multicentric Evaluation of SeeGene Allplex Real-Time PCR Assays Targeting 28 Bacterial, Microsporidal and Parasitic Nucleic Acid Sequences in Human Stool Samples. Diagnostics.

[64] Loderstädt U., Hagen R.M., Hahn A., Frickmann H. (2021). New Developments in PCR-Based Diagnostics for Bacterial Pathogens Causing Gastrointestinal Infections-A Narrative Mini-Review on Challenges in the Tropics. Trop. Med. Infect. Dis..

[65] Hoffmann T., Hahn A., Verweij J.J., Leboulle G., Landt O., Strube C., Kann S., Dekker D., May J., Frickmann H. (2021). Differing Effects of Standard and Harsh Nucleic Acid Extraction Procedures on Diagnostic Helminth Real-Time PCRs Applied to Human Stool Samples. Pathogens.

[66] Kyei G., Ayi I., Boampong J.N., Turkson P.K. (2015). Sero-Epidemiology of *Toxocara Canis* Infection in Children Attending Four Selected Health Facilities in the Central Region of Ghana. Ghana Med. J..

